# Copy‐number intratumor heterogeneity as high‐risk feature of stage II colon cancer^†^

**DOI:** 10.1002/path.5919

**Published:** 2022-05-23

**Authors:** Tom van den Bosch, Daniël M Miedema, Louis Vermeulen

**Affiliations:** ^1^ Amsterdam UMC location University of Amsterdam, Cancer Center Amsterdam & Amsterdam Gastroenterology Endocrinology Metabolism, Center for Experimental and Molecular Medicine Amsterdam The Netherlands; ^2^ Oncode Institute Amsterdam The Netherlands

**Keywords:** stage II colon cancer, biomarkers, predictive modeling, intratumor heterogeneity

## Abstract

Overall, the prognosis of patients suffering from stage II colon cancer is relatively favorable. However, a proportion of patients develop a recurrence following surgery. Clinical and histopathological properties that identify high‐risk patients are of limited value and better biomarkers are urgently required. In a recent issue of *The Journal of Pathology*, Lahoz *et al* proposed that copy‐number‐based biomarkers could be employed for patient stratification. The authors studied copy‐number alterations (CNAs) at the genomic scale by measuring the total CNA load (the aberrant genome fraction), and at a smaller scale by identifying common arm‐ or cytoband‐level alterations. Both the overall CNA load and specific chromosomal regions were associated with an increased risk of recurrence. Most interestingly, it was demonstrated that copy‐number intratumor heterogeneity, as defined by subclonal CNAs, is associated with poor disease outcome. This study demonstrates that structural genomic aberrations are promising biomarkers for patient stratification in early colon cancer. © 2022 The Authors. *The Journal of Pathology* published by John Wiley & Sons Ltd on behalf of The Pathological Society of Great Britain and Ireland.

Around 10–15% of stage II colon cancer patients relapse within 5 years after surgery [1]. Treatment of all stage II colon cancer patients with adjuvant chemotherapy is associated with only a minimal overall reduction in recurrence rates [2], not outweighing the side effects and risks associated with treatment itself. Ideally, only patients with a high risk of relapse would receive adjuvant therapy to reduce the recurrence rate, while patients that are less likely to relapse should be spared exposure to chemotherapy. Selection of high‐risk stage II patients based on clinicopathological features is suboptimal. Currently, these cancers are identified based on bowel obstruction or perforation, poor differentiation grade, lymphovascular invasion, insufficient lymph node retrieval, and advanced T stage. Of these features, T4 stage seems to be the most important determinant, but the vast majority of stage II T4 cancers do not develop a recurrence, even without adjuvant therapy. With respect to molecular markers, *BRAF* ^V600E^ mutations (high‐risk) and microsatellite instability (MSI, low‐risk) are of value, but these only comprise small groups of patients [3]. Therefore, there is an urgent need to develop novel biomarkers that can stratify stage II cancers into distinct risk groups, and ideally predict benefit from adjuvant therapy.

In a recent and very relevant study, Lahoz *et al* investigated whether chromosomal copy‐number alterations (CNAs) can predict recurrence rates in early colon cancer [4]. Their cohort consisted of 84 stage II colon cancer patients who did not receive adjuvant therapy, and that was enriched for cases that developed a recurrence (*n* = 38, 45%). The authors first noted differences between the recurrent and nonrecurrent patient groups in common clinical characteristics and previously identified histopathological biomarkers. Lahoz *et al* [4] further studied genetic biomarkers, including somatic mutations, specific CNAs, genome‐wide CNA load, and copy‐number intratumor heterogeneity (ITH).

In line with earlier studies, Lahoz *et al* [4] found a significant association of recurrence with reported high‐risk markers, including poor differentiation grade, tumor budding, and lymphovascular invasion. In addition, a low CD8+ lymphocyte infiltration rate predicted an increased probability of recurrent disease [5]. The authors further found somatic mutations in the *SOX9*, *NOTCH1*, and *SYNE1* genes to be associated with a higher risk of recurrence. Specific chromosomal alterations associated with a higher risk were found on chromosomes 13q, 17p, and 17q, which further indicates a possible impact of the *SOX9* gene located at locus 17q24.3. Previous studies have also mentioned chromosome 13q and the genes *DIS3* and WNT‐related genes *CDX2* and *CDK8* at this chromosome arm to be related to colorectal cancer progression [6, 7, 8]. More generally, total CNA load was also found to increase the risk of recurrence, in accordance with previous results in a pan‐cancer study that measured whole‐genome copy‐number load [9].

ITH is a biomarker that has shown prognostic relevance in many malignancies in recent years, but until now no specific results were reported on the prognostic value of copy‐number ITH in early colon cancer. Lahoz *et al* found that a high number of subclonal CNAs was associated with a higher risk of recurrence in both a univariate and multivariable setting [4]. The results of that study are in line with a recent study that showed copy‐number ITH to be predictive of survival in many different cancer types and tumor stages [10]. Interestingly, mutational ITH showed no relation to risk of recurrence in the current study. This indicates that ITH at the copy‐number level is a robust biomarker and more related to poor disease outcome as compared to single nucleotide variants, which could provide an insight into the underlying mechanism.

Copy‐number ITH is thought to be driven by ongoing chromosomal instability (CIN) [11], which causes constant creation of new subclones with different karyotypes and phenotypes. Indeed, measurements of copy‐number ITH correlate with measurements of CIN [10]. Higher copy‐number ITH could reflect a higher rate of evolution and higher phenotypic heterogeneity within tumors [11]. High phenotypic heterogeneity and accelerated evolution allows a tumor to explore a wider selection of fitness landscapes, which might increase the ability to evade the immune system and to deal with other selective pressures. Phenotypic heterogeneity typically increases the probability that subclones can successfully seed to different microenvironments (Figure 1). A higher likelihood of migration and metastatic seeding could be a viable explanation for the relation between copy‐number ITH and risk of recurrence, as microscopic metastases might already be present at the time of surgery. It cannot be excluded that the lack of relation between mutational ITH and recurrence is due to the limited gene panel that was assessed, but it could also imply that copy‐number ITH has a more pronounced impact on phenotypic heterogeneity than mutational ITH. In this respect, MSI tumors are of interest, as they generally have high mutational ITH and low copy‐number ITH and low CNA load and are well known to have a better prognosis than microsatellite stable (MSS) tumors in low‐stage colon cancer [3]. This further enforces copy‐numbers to be the determining ITH factor in relation to recurrence risk.

**Figure 1:**
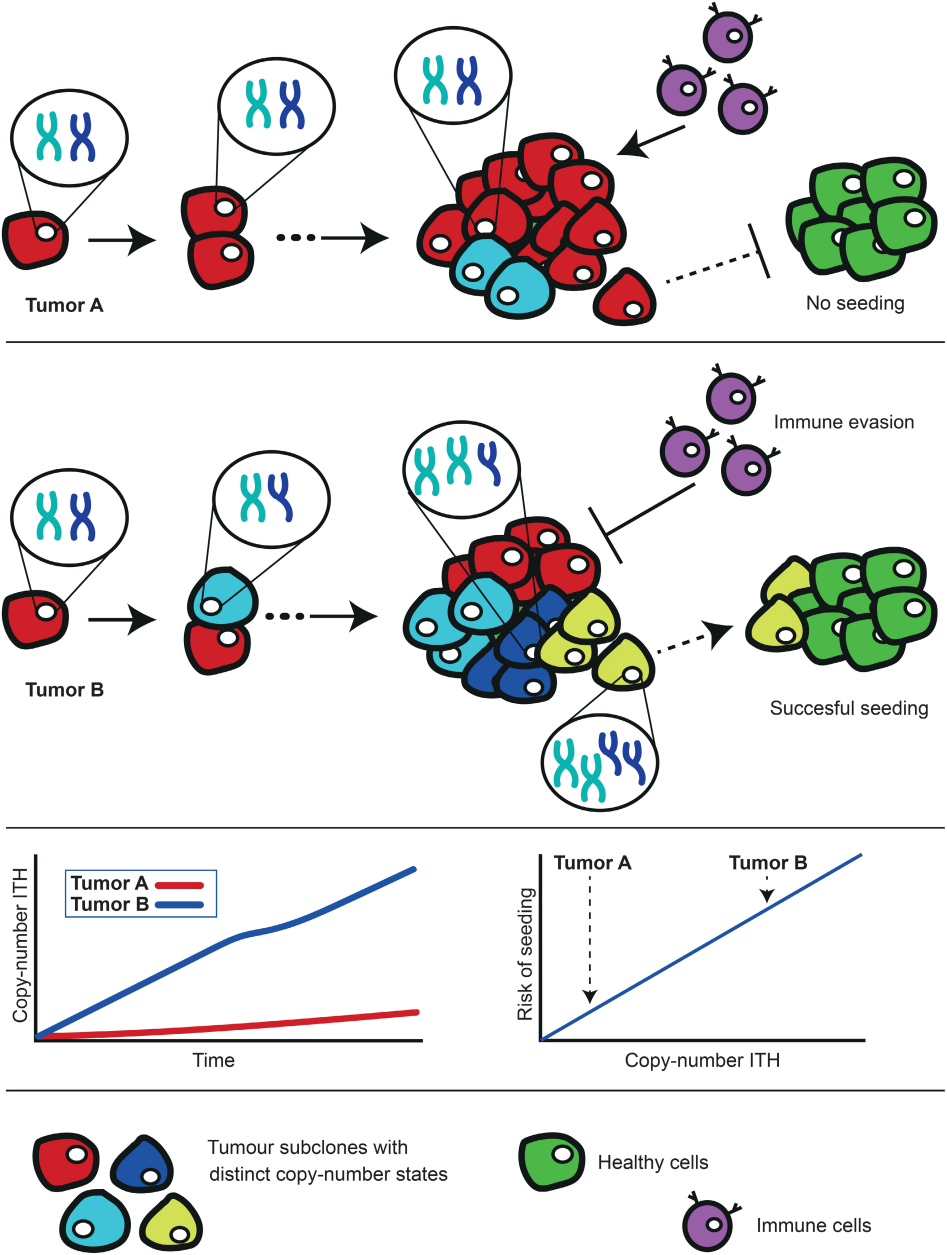
Copy‐number intratumor heterogeneity increases risk of recurrence. Copy‐number intratumor heterogeneity enhances the immune evasion of a cancer and increases the probability of subclones successfully migrating to and seeding in other microenvironments, increasing the risk of recurrence after surgery.

Altogether, the study by Lahoz *et al* [4] provided new key insights into molecular risk prediction in stage II colon cancer, but also acknowledges that further research is needed. Specifically, copy‐number ITH was found to be a marker for disease recurrence that should be validated in additional cohorts. It would also be interesting to incorporate less invasive methods, such as liquid biopsies to measure copy‐number ITH in circulating tumor DNA that could aid in determining ITH or the presence of possibly metastatic subclones. Importantly, the current results indicate a prognostic value of CNAs and follow‐up research should investigate the benefit of adjuvant chemotherapy in this patient group. As colon cancer is a heterogeneous disease with multiple subgroups that could influence therapy efficacy [12], it is perceivable that the unique biology that is associated with increased copy‐number ITH also selects for therapy‐resistant cancers. Therefore, demonstrating the predictive value of biomarkers for high‐risk stage II colon cancers will ultimately depend on randomized clinical trial data.

## Author Contributions Statement

All authors were responsible for writing and approving the final version of the article.

## References

[1] Tournigand C , de Gramont A . Chemotherapy: is adjuvant chemotherapy an option for stage II colon cancer? Nat Rev Clin Oncol 2011; 8: 574–576. 21912416 10.1038/nrclinonc.2011.139

[2] André T , de Gramont A , Vernerey D , *et al*. Adjuvant fluorouracil, leucovorin, and oxaliplatin in stage II to III colon cancer: updated 10‐year survival and outcomes according to BRAF mutation and mismatch repair status of the MOSAIC study. J Clin Oncol 2015; 33: 4176–4187. 26527776 10.1200/JCO.2015.63.4238

[3] Dienstmann R , Villacampa G , Sveen A , *et al*. Relative contribution of clinicopathological variables, genomic markers, transcriptomic subtyping and microenvironment features for outcome prediction in stage II/III colorectal cancer. Ann Oncol 2019; 30: 1622–1629. 31504112 10.1093/annonc/mdz287 PMC6857614

[4] Lahoz S , Archilla I , Asensio E , *et al*. Copy‐number intratumor heterogeneity increases the risk of relapse in chemotherapy‐naive stage II colon cancer. J Pathol 2022; 257: 68–81. 35066875 10.1002/path.5870 PMC9790656

[5] Pagès F , Mlecnik B , Marliot F , *et al*. International validation of the consensus Immunoscore for the classification of colon cancer: a prognostic and accuracy study. Lancet 2018; 391: 2128–2139. 29754777 10.1016/S0140-6736(18)30789-X

[6] de Groen FL , Krijgsma O , Tijsse M , *et al*. Gene‐dosage dependent overexpression at the 13q amplicon identifies DIS3 as candidate oncogene in colorectal cancer progression. Genes Chromosomes Cancer 2014; 53: 339–348. 24478024 10.1002/gcc.22144

[7] Salari K , Spulak ME , Cuff J , *et al*. CDX2 is an amplified lineage‐survival oncogene in colorectal cancer. Proc Natl Acad Sci U S A 2012; 109: E3196–E3205. 23112155 10.1073/pnas.1206004109 PMC3503165

[8] Firestein R , Bass AJ , Kim SY , *et al*. CDK8 is a colorectal cancer oncogene that regulates beta‐catenin activity. Nature 2008; 455: 547–551. 18794900 10.1038/nature07179 PMC2587138

[9] Taylor AM , Shih J , Ha G , *et al*. Genomic and functional approaches to understanding cancer aneuploidy. Cancer Cell 2018; 33: 676–689.e3. 29622463 10.1016/j.ccell.2018.03.007 PMC6028190

[10] van Dijk E , van den Bosch T , Lenos KJ , *et al*. Chromosomal copy number heterogeneity predicts survival rates across cancers. Nat Commun 2021; 12: 3188. 34045449 10.1038/s41467-021-23384-6 PMC8160133

[11] Sansregret L , Vanhaesebroeck B , Swanton C . Determinants and clinical implications of chromosomal instability in cancer. Nat Rev Clin Oncol 2018; 15: 139–150. 29297505 10.1038/nrclinonc.2017.198

[12] Ten Hoorn S , de Back TR , Sommeijer DW , *et al*. Clinical value of consensus molecular subtypes in colorectal cancer: a systematic review and meta‐analysis. J Natl Cancer Inst 2022; 114: 503–516. 34077519 10.1093/jnci/djab106 PMC9002278

